# Polysaccharide-Based Multifunctional Hydrogel Bio-Adhesives for Wound Healing: A Review

**DOI:** 10.3390/gels9020138

**Published:** 2023-02-06

**Authors:** Jiahao Yang, Shige Wang

**Affiliations:** School of Materials and Chemistry, University of Shanghai for Science and Technology, No. 516 Jungong Road, Shanghai 200093, China

**Keywords:** wound healing, hydrogel bio-adhesives, multifunctional, polysaccharides

## Abstract

Wound healing is a long-term and complex biological process that involves multiple hemostasis, inflammation, proliferation, and remodeling stages. In order to realize comprehensive and systematic wound management, appropriate wound treatment bio-adhesives are urgently needed. Hydrogel bio-adhesives have excellent properties and show unique and remarkable advantages in the field of wound management. This review begins with a detailed description of the design criteria and functionalities of ideal hydrogel bio-adhesives for wound healing. Then, recent advances in polysaccharide-based multifunctional hydrogel bio-adhesives, which involve chitosan, hyaluronic acid, alginate, cellulose, dextran, konjac glucomannan, chondroitin sulfate, and other polysaccharides, are comprehensively discussed. Finally, the current challenges and future research directions of polysaccharide-based hydrogel bio-adhesives for wound healing are proposed to stimulate further exploration by researchers.

## 1. Introduction

The management and healing of different types of wounds (e.g., skin injury [1], heart injury [2], muscle injury [3], gastrointestinal injury [4], corneal injury [5], lung injury [6], etc.) have become a significant problem costing tens of billions of dollars worldwide. Even though sutures and staples have been considered the “gold standard” for wound closure, they are not suitable for all kinds of wounds. In addition, due to their disadvantages, such as secondary damage to the tissue and risk of infection, sutures and staples are not conducive to wound healing and the restoration of tissue structure to a certain extent [7]. In contrast, bio-adhesives are less invasive and more effective [8]. Therefore, the development of bio-adhesives for wound healing has received ever-increasing attention.

Biocompatible hydrogel is a class of material with three-dimensional porous structures, which is similar to the natural extracellular matrix (ECM) [9]. They are highly flexible and able to adapt to the shapes of wounds and hold large volumes of biological fluids to keep the wound moist. In addition, hydrogels can effectively deliver drugs and bioactive substances to target sites [10,11]. Moreover, they have sufficient mechanical strengths and good self-healing properties, which affords their durable and sustainable usage as bio-adhesives [12]. Together with their antibacterial and antioxidant properties, hydrogel bio-adhesives have been considered promising candidates for wound-healing dressings [13,14].

Polysaccharides are typically derived from plants and animals [15]. Polysaccharide-based hydrogel possesses excellent biocompatibility and biodegradability and has promising application potential in the biomedical and other fields [16,17]. In recent years, many significant achievements have been made in the construction of polysaccharide-based hydrogel bio-adhesives. Representative substances of polysaccharides include chitosan (CS) [18], dextran [19], cellulose [20], hyaluronic acid (HA) [21], alginate [22], konjac glucomannan (KGM) [23], chondroitin sulfate [24], and so on. A limitation of polysaccharide-based hydrogel bio-adhesives is their insufficient mechanical property, which leads to the failure of cohesion and limits their application prospects in the field of wound healing [25]. 

Generally, chemical modifications, which can synergistically improve the mechanical properties of hydrogel bio-adhesives through multiple cross-linking reactions, have been carried out towards various chemical groups (e.g., hydroxyl, carboxyl, amino, sulfhydryl, etc.) of polysaccharides [26,27]. The degree of modification and cross-linking, and the type of chemical bonds significantly affect the mechanical strengths of polysaccharide-based hydrogel bio-adhesives. Covalent cross-linking (e.g., free radical chain polymerization, click chemistry, etc.) has been frequently used to enhance the structural stability of hydrogel bio-adhesives [28]. Excitingly, chemical modifications may bring new functionalities to hydrogel bio-adhesives. For example, dynamic covalent cross-linking, such as Schiff base reaction, boronate ester bond cross-linking, and disulfide bond cross-linking, can introduce self-healing properties into the hydrogel bio-adhesives [29]. Furthermore, physical interactions (e.g., hydrogen bonding, metal-ligand coordination cross-linking, etc.) can also substantially contribute to enhancing the flexibility and endowing polysaccharide-based hydrogel bio-adhesives with injectability and self-healing properties [30].

**Scheme 1:**
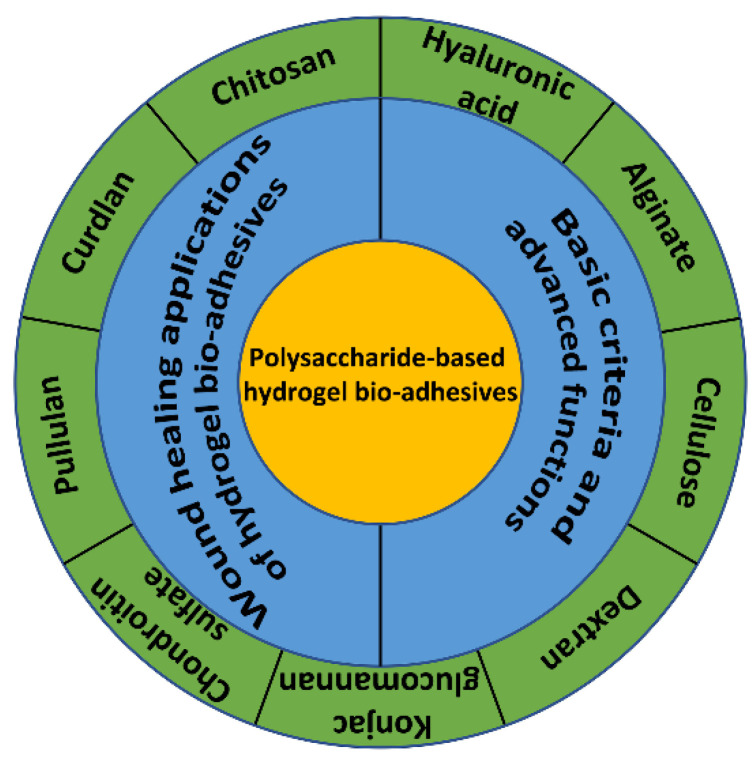
Polysaccharide-based multifunctional hydrogel bio-adhesives and wound-healing applications.

The overall goal of this review is to introduce the recent developments of polysaccharide-based hydrogel bio-adhesives, focusing on the multiple functions of this biomedical material. In addition, the design criteria and functionalities of ideal hydrogel bio-adhesive systems for wound healing are briefly summarized, and the working principles of the hydrogel bio-adhesives are described in detail. Finally, the current challenges of polysaccharide-based hydrogel bio-adhesives in wound healing applications are discussed, and prospects are given for their future developments (Scheme 1). Most of the reviews that have been published mainly described various polymers (natural and synthetic) that can be used in the preparation of hydrogels and their applications [31,32]. However, few reviews summarize the design criteria and functionality of hydrogel bio-adhesives.

## 2. Design Criteria and Functionalities of Hydrogel Bio-Adhesives for Wound Healing

**Figure 1:**
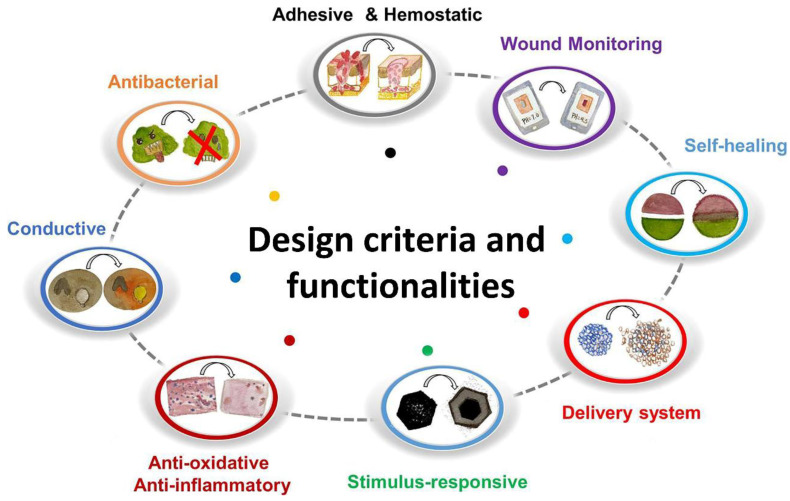
Design criteria and functionalities of hydrogel bio-adhesives for wound healing. Reprinted with permission from ref [33]. Copyright 2021 American Chemical Society.

Until now, diverse hydrogel bio-adhesives have been developed with various biomedical functions to meet the increasing clinical requirements for wound healing. The basic design criteria and advanced functions are summarized in the section (Figure 1).

### 2.1. Design Criteria

#### 2.1.1. Hemostasis

The first stage of wound healing is hemostasis; therefore, hydrogel bio-adhesives for wound healing should have hemostatic properties [34,35]. Bleeding wounds could be closed by hydrogel bio-adhesives with strong wet adhesion properties and suitable mechanical strength. For blood pressure in the heart and vascular injuries, hydrogel bio-adhesives should have the mighty mechanical property to withstand high burst pressure [36]. Additionally, substances with hemostatic activity (e.g., clay, cationic polysaccharides, polypeptides, etc.) could be incorporated into the hydrogel bio-adhesive systems to enhance their hemostatic properties [37]. The hemostatic properties of the hydrogel bio-adhesives are typically evaluated by in vitro coagulation tests; blood cells and platelets adhesion activation tests; and in vivo experiments, such as liver hemostasis, cardiac hemostasis, and tail docking hemostasis.

#### 2.1.2. Wound Closure

Wound closure is the primary criterion of hydrogel bio-adhesives, giving them great potential as alternatives to traditional sutures [38]. Wound closure may further arrest bleeding, maintain the structures and functions of tissues, and prevent the intrusion of bacteria and foreign bodies. For wounds on different organs, the wound closure of hydrogel bio-adhesives also needs to be adjusted accordingly. For example, for the closure of lung wounds, hydrogel bio-adhesives should prevent gas leakage [39]. However, for the closure of skin or heart wounds, the hydrogel bio-adhesives should accommodate the movement of the tissues and ensure wound closure [39].

#### 2.1.3. Adhesion

Good adhesive strength is a necessary property of hydrogel bio-adhesives [40]. Hydrogel bio-adhesives with strong adhesion can provide physical barriers to impede bleeding and prevent the invasion of bacteria and foreign bodies. Notably, the interfacial barrier formed by blood and tissue fluid on the wound surface can hinder the adhesion between biological tissues and hydrogel bio-adhesives. Therefore, wet adhesion of hydrogel bio-adhesives needs to be developed to overcome the interference of body fluids through different strategies, such as hydrophobic interactions and mechanical interlocking [41,42]. In addition, other factors that affect adhesive performance, such as frequent movement and the pH of certain specific wounds, also need to be overcome [43]. Currently, the adhesive strength of hydrogel bio-adhesives can be evaluated by tissue adhesion demonstration, lap shear test, peel test, underwater adhesion test, burst pressure test, and so on.

#### 2.1.4. Biocompatibility

The biocompatibility of biomaterials involves cytocompatibility, histocompatibility, hemocompatibility, and so on [44]. Hydrogel bio-adhesives with good biocompatibility should be characterized by toxicity-free, low hemolysis rate, low inflammatory response, low immunogenicity, and low risk of infection and cancerization [45]. Biological toxicity could be avoided by controlling the molecular weight of the components and selecting appropriate substances; in addition, immune elimination responses can be reduced by controlling the appropriate hydrophilicity and hydrophobicity of the hydrogel bio-adhesives [46,47]. Moreover, the evaluation of hemocompatibility is also necessary for bleeding wounds. Typically, in vivo implantation assays and metabolic assays are used to evaluate the histocompatibility and systemic toxicity of the hydrogel bio-adhesives [48].

#### 2.1.5. Biodegradability

Biodegradability is one of the crucial properties of hydrogel bio-adhesives. The hydrogel bio-adhesives with good biodegradability will be degraded by enzymes or body fluids in the organism to avoid secondary damage to the body [49]. Moreover, the biodegradation rate of hydrogel bio-adhesives is controllable through the selection and modification of ingredients and the adjustment of counterpart ratios. At different wound sites, hydrogel bio-adhesives with corresponding degradable properties have the enhanced ability to promote wound repair [39]. Meanwhile, the degradation products should be non-toxic or low-toxic and excreted through the body’s metabolic pathways. 

### 2.2. Advanced Functions

#### 2.2.1. Self-Healing

Hydrogel bio-adhesives are susceptible to damage due to external shear forces or tissue activity, leading to a shortened lifespan, invasion of external bacteria, and risk of infection. As a smart material, self-healing hydrogel bio-adhesive is able to repair the damage, and maintain the integrity of structure and function [50]. The self-healing properties of hydrogel bio-adhesives rely on dynamic and reversible interactions, a large proportion of which are typically Schiff base (imine) structure and hydrogen bonds [51]. Furthermore, the mechanical properties of the hydrogel bio-adhesives should match the target wound tissues to prevent exfoliation and maintain the stability of the physical barrier [39].

#### 2.2.2. Antibacterial

Bacterial infection, which may cause a persistent inflammatory response at the wound site and lead to delayed wound healing and even severe complications, is a great challenge during wound healing [52]. Various strategies, such as the selection of polymers with antibacterial activities and the loading of antibacterial drugs, inorganic metal nanoparticles, photothermal substances, and nanozymes, have been developed to endow hydrogel bio-adhesives with good antibacterial abilities [53,54,55,56,57,58]. Among them, the abuse of antibacterial drugs may lead to bacterial resistance; inorganic metal antibacterial agents could cause biological toxicity and long-term retention; and photothermal therapy and nanozymes have a longer clinical translation time and uncertain translation prospects. Therefore, polymers with inherent antibacterial activity have been the subject of intense research [59]. The most commonly used methods for in vitro antibacterial tests are the agar diffusion method and the minimum inhibitory concentration determination test. Common in vivo skin wound infection assays are frequently used to evaluate the in vivo antimicrobial capacities of hydrogel bio-adhesives.

#### 2.2.3. Anti-Inflammatory and Antioxidant

Excessive wound inflammation, accompanied by the generation of massive reactive oxygen species (ROS) (such as hydroxyl radicals, hydrogen peroxide, superoxide anion, nitric oxide, etc.), tends to cause cellular damage by triggering deoxyribonucleic acid (DNA) oxidation, protein oxidation, and lipid peroxidation [60]. Free radicals are able to be captured and neutralized by substances with antioxidant activities to downregulate inflammatory responses. Consequently, antioxidant hydrogel bio-adhesives capable of controlling inflammation are highly admirable for wound healing. Recently, polyphenolic antioxidants with good stability (such as tea polyphenols, curcumin, etc.) are widely used in the design of wound dressings [61,62]. Dopamine (DA) belongs to the class of catecholamine neurotransmitters and shows good antioxidant properties. In addition, it was reported that the Bletilla striata polysaccharide may also impart anti-inflammatory properties to the hydrogel bio-adhesives [28]. Typically, certain probes, such as α, α-Diphenyl-β-picrylhydrazine (DPPH), and 2,2-Diazo-bis (3-ethyl-benzothiazole-6-sulfonic acid) diammonium salt (ABTS), are also used to examine the antioxidant activity of hydrogel bio-adhesives [63,64].

#### 2.2.4. Loading and Controlled Delivery

Hydrogel bio-adhesives with loose porous structures could load a variety of effective substances and slowly release them at the wound site [65]. A study has shown that many factors, including cellular interactions, cytokines, growth factors (GFs), chemokines, etc., favor the wound healing [33]. According to proven studies, local delivery of exogenous cells (e.g., adipose-derived stem cells (ADSCs), bone marrow mesenchymal stem cells (BMSCs), human umbilical cord mesenchymal stem cells, fibroblasts, and human microvascular endothelial cells (HMECs), etc.) or GFs (e.g., epidermal growth factor (EGF), fibroblast growth factor (FGF), vascular endothelial growth factor (VEGF) and keratinocyte growth factor (KGF), etc.) are able to synergize with endogenous factors to accelerate wound repair [66,67,68,69,70,71]. Moreover, some functional proteins and peptides, such as feather keratin, neurotensin, and the laminin mimetic peptide SIKVAV, could also be loaded by wound dressings to exert specific effects [72,73,74].

#### 2.2.5. Stimulus-Response Property

Stimuli-responsive hydrogel bio-adhesives are capable of producing changes in shapes, properties, and sizes in response to external stimuli (e.g., pH, temperature, light, etc.) [75]. For example, some thermo-responsive components are fluid liquids at low temperatures, while rapidly transform into hydrogels at normal body temperatures [76]. It has been reported that poly (N-isopropyl acrylamide), polyethylene glycol (PEG), and collagen have been used to make heat-sensitive hydrogel bio-adhesives [77,78]. Many pH-responsive hydrogel bio-adhesives have been widely developed. For instance, in response to low pH, the coordination between tannins and metal ions decreased, which released tannins to fight bacteria and eliminate inflammation [39]. In a study, photo-responsive cationic polyethyleneimine/anionic pectin hydrogel bio-adhesives with good controllability, photothermal antibacterial, and light-responsive release capacities were established [79].

#### 2.2.6. Electrical Conductivity

The positive charge at the wound site and the negative charge in normal skin interact to form a system named skin battery [80]. The resulting endogenous electric field is able to guide cells to migrate into the wound site and promote wound healing [80]. Therefore, the migration of neutrophils, macrophages, and keratinocytes is promoted by applying an external current to the hydrogel bio-adhesives by mimicking the endogenous current [81]. As electrical signaling molecules, integrins, EGF receptors, PI3 kinase/Pten (phosphoinositide 3-kinases/phosphatase and tensin homologue), and V-ATPase H^+^ pump also play essential roles in the system [80]. Conductive polymers, such as polypyrrole, polythiophene, and poly(3,4-ethylenedioxythiophene), could be mixed with natural polysaccharides to synthesize electroactive hydrogel bio-adhesives with electrical conductivity [82,83].

#### 2.2.7. Wound Monitoring

The process of wound healing is complex, and the wound microenvironment is in a dynamically changing status. Therefore, fabricating wound-monitoring hydrogel bio-adhesives to monitor and manage wounds is beneficial for enhanced wound care [84]. For example, Zhu et al. designed a multifunctional zwitterionic hydrogel bio-adhesive that could monitor the glucose concentration (in the range of 0.1–10 × 10^−3^ M) and the pH (in the range of 4–8), which was beneficial to observe and control the wound conditions of diabetic patients [85].

## 3. Multifunctional Hydrogel Bio-Adhesives Based on Polysaccharides

Polysaccharides are sugar chains composed of monosaccharide or disaccharide repeating units, which are linked by glycosidic bonds. Polysaccharides are widely used in hydrogel bio-adhesive fabrication because of their satisfactory biocompatibility, biodegradability, unique biological activity, excellent pro-healing properties, easy physical and chemical modification, suitable viscoelasticity, and rich sources (Table 1). 

### 3.1. Chitosan (CS)

CS is produced by the chemical or enzymatic deacetylation of chitin, and the repeating units that constitute CS are glucosamine and N-acetyl-D-glucosamine [104,105]. CS is a kind of cationic polysaccharide [106]. CS possesses biocompatibility, biodegradability, and mucoadhesive property. After contact with blood, -NH_2_ groups on the CS molecular chain can be partially protonated to -NH_3_^+^, endowing CS with good antibacterial activity [107,108]. These abundant -NH_3_^+^ groups are able to promote the rapid aggregation and adhesion of platelets, red blood cells, and proteins at the injured site through electrostatic interactions, resulting in strong blood coagulation and the formation of thrombus [109]. Furthermore, CS may promote the expression of GPIIb/IIIa receptors on the platelet surface and the mobilization of [Ca^2+^]_i_ to further induce platelet aggregation [110]. In addition, it has been found that CS may not only regulate the secretion of inflammatory mediators but also enhance the function of inflammatory cells (such as polymorphonuclear leukocytes and macrophages, etc.) during the inflammatory stage to promote granulation tissue formation [111]. More interestingly, CS is competent in promoting the proliferation of fibroblasts, neovascularization, collagen deposition, and the production of HA to accelerate wound repair [112].

(1) CMCS

Different chemical modifications (e.g., carboxymethylation, quaternization, catechol grafting, etc.) have been devoted to improving the water solubility of CS [113,114]. A successful attempt is to transform CS into carboxymethyl CS (CMCS). Owing to the presence of -NH_3_^+^ and -COO^−^, CMCS gains enhanced water solubility in a neutral solution and inherits the advanced properties of pristine CS [115]. For example, a melatonin-containing injectable CMCS-based hydrogel bio-adhesive was prepared by Hou et al. [86]. Melatonin is able to regulate the release of inflammatory mediators, promote the migration and proliferation of cells, and accelerate collagen deposition. The wound-healing effect of the hydrogel bio-adhesive was evaluated on the full-thickness skin wound model of a rat. The results demonstrated that the melatonin-loaded CMCS-based hydrogel bio-adhesive reduced the inflammatory response and induced granulation tissue formation through multiple potent effects and biological events.

(2) Quaternized chitosan (QCS)

The substitution reaction between the amino groups of CS and glycidyl trimethyl ammonium chloride introduces quaternary ammonium groups into the CS chains and increases the number of positive charge centers of CS. These positive charge centers promote the adhesion and aggregation of blood cells and enhance the antibacterial activity and hydrophilicity of CS [69,116]. In a study, Gao and colleagues designed a multifunctional dual colorimetry-integrated hydrogel bio-adhesive to effectively monitor pH and wound conditions in real-time. The hydrogel bio-adhesive was composed of polyacrylamide (PAM), quaternized CS (QCS), carbon quantum dots (CQDs), and phenol red [87]. Compared with CS, QCS has more robust broad-spectrum antibacterial activities and enhanced hemostatic activities. The use of CQDs and phenol red enabled accurate, highly responsive, and reversible pH indication, which effectively reflected dynamic wound conditions under visible and UV light [87]. Meanwhile, this hydrogel bio-adhesive could monitor the process of wound healing remotely and intelligently through a smartphone. However, the biotoxicity of quaternary ammonium groups should be taken into consideration [117].

(3) Thiolated CS

Thiol groups could be introduced into the CS through the modification of primary amine groups to enhance water solubility, mucoadhesive properties, and hemostatic activity [118]. In the study by Feng et al., a thiolated CS, CS-4-thiobutylamidine (CS-TBA) conjugate was combined with β-glycerophosphate disodium (β-GP) to form an injectable thermosensitive hydrogel bio-adhesive for the repair of irregular wounds [88]. This novel CS-TBA/hydroxyapatite/β-GP system could be converted into a biocompatible and biodegradable hydrogel bio-adhesive at physiological temperature, which exhibited good rheology as well as a higher storage modulus (G’) and loss modulus (G”). In addition, it had a lower protein release rate through the action of disulfide bonds.

(4) Catechol-modified CS

**Figure 2:**
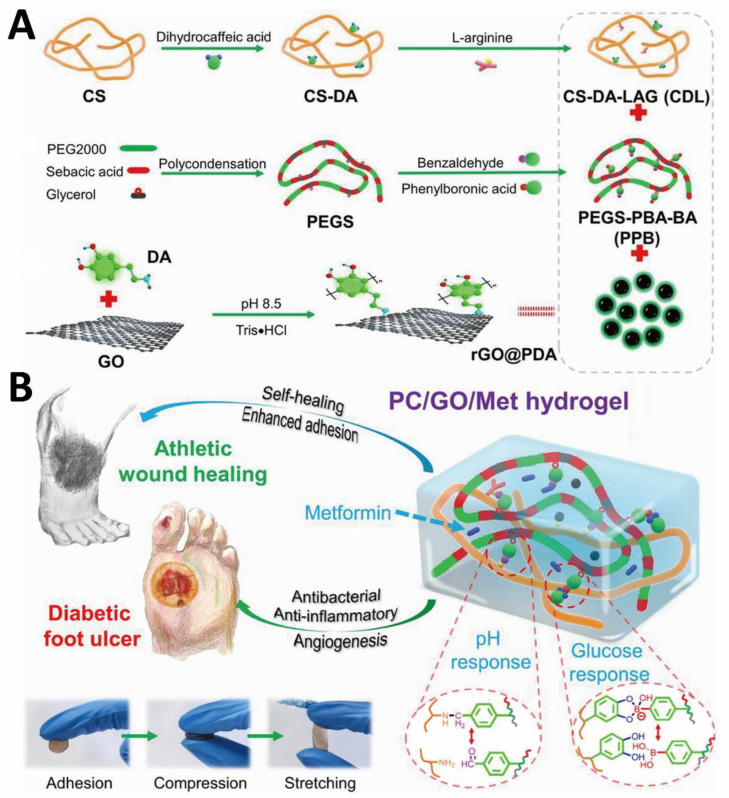
CS-based hydrogel bio-adhesive: schematic diagram of (**A**) preparation and (**B**) application of CS-based hydrogel bio-adhesive. Reprinted with permission from ref [90]. Copyright 2022 American Chemical Society.

Catechol groups exhibit strong and versatile adhesion capabilities on different surfaces [119]. Although photo-crosslinked polysaccharide-based hydrogel bio-adhesives have good biological properties, they are insufficient in terms of adhesion and mechanical properties. To solve these problems, Wang et al. prepared injectable photo-crosslinked hydrogel bio-adhesives with adjustable gelation time and simple gelation conditions. In this study, the hydrogel formation was based on the polymerization of unsaturated bonds and catechol-Fe^3+^ chelation [89]. The double networks and crosslinks endowed the hydrogel bio-adhesives with improved mechanical properties. The -NH_3_^+^ of CS and the quinone group formed by the oxidation of catechol synergistically enhanced the antibacterial properties of hydrogel bio-adhesives. Moreover, catechol-Fe^3+^ chelation provided strong adhesion and cohesive strength. In another research, Guo and colleagues developed a phenylboronic acid and benzaldehyde bifunctional PEG-co-polyglycerol sebacic acid/dihydrocaffeic acid and arginine co-grafted CS hydrogel bio-adhesive for treating diabetic foot (Figure 2A) [90]. With double dynamic bonds, the as-designed hydrogel bio-adhesive possessed good self-healing properties, strong adhesion, electrical conductivity, antibacterial, and hemostatic activities. Such a multifunctional hydrogel bio-adhesive is able to release metformin in response to low pH and high blood glucose levels of chronic athletic type II diabetic foot (Figure 2B). The catechol structure of dihydrocaffeic acid provided good antioxidant properties and tissue adhesion, and L-arginine disrupted the hydration layer at the interface to enhance adhesion. The electrical conductivity originated from the polydopamine (PDA)-coated reduced graphene oxide (rGO@PDA), which was directly doped within the hydrogel precursor solution.

(5) Hydrophobically modified CS

The introduction of hydrophobic groups is able to enrich the functionalities of CS. For example, the reaction between a dodecyl aldehyde and -NH_2_ enhanced its hydrophobicity [120]. Dodecyl is capable of penetrating the cell membrane with its tail to promote the accumulation of red blood cells [120]. Additionally, excessive swelling may decrease the stability of hydrogel bio-adhesives and damage surrounding tissues; hydrophobically modified CS has been used to prepare non-swelling hydrogel bio-adhesive. For instance, Li et al. designed an injectable biocompatible pentenyl CS-based hydrogel bio-adhesive [91]. Through one-step N-acylation, UV-cross-linkable n-pentenyl groups were introduced into the backbone to achieve rapid in situ gelation. As a short hydrophobic alkyl chain, n-pentenyl could break a large number of hydrogen bonds and form a hydrophobic polymer network to repel water and counteract swelling.

### 3.2. Hyaluronic Acid (HA)

HA is a non-sulfated glycosaminoglycan and natural anionic polysaccharide [121,122]. Repeating disaccharide units comprising β-D-glucuronic acid and N-acetyl-D-glucosamine are linked by alternating β-1,3 and β-1,4 glycosidic linkages to form HA. As the main component of ECM in human tissues, HA possesses non-immunogenicity, excellent biocompatibility, and biodegradability. In addition, HA has good hydrophilicity, moisturizing ability, and outstanding gel-forming property. It has been found that HA is able to promote blood clot formation, interleukin expression, new blood vessel formation, and the migration and proliferation of keratinocytes and fibroblasts [123]. Additionally, HA is able to reduce inflammatory cell infiltration, regulate the activity of GFs, control collagen deposition, and improve granulation tissue formation and re-epithelialization [124]. 

**Figure 3:**
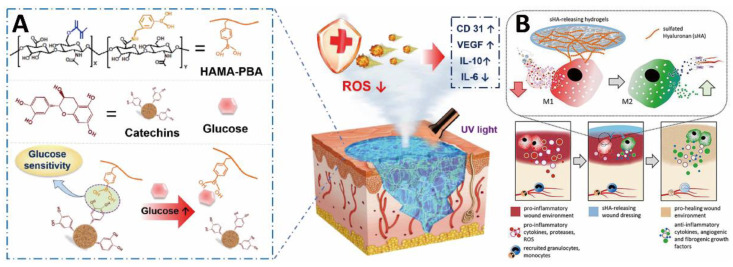
HA-based hydrogel bio-adhesives: (**A**) schematic diagram illustrating the glucose-responsive antioxidant hydrogel bio-adhesive for diabetic wound healing. Reprinted with permission from ref [57]. Copyright 2022 Elsevier. (**B**) Schematic illustration of modulation of inflammatory macrophage activity by hydrogel bio-adhesive. Reprinted with permission from ref [94]. Copyright 2021 Elsevier.

Research on antioxidant HA-based hydrogel bio-adhesives for the treatment of chronic diabetic wounds has yielded remarkable results. As a paradigm, Wu et al. developed a glucose-responsive antioxidant hydrogel bio-adhesive through the polymerization of phenylboronic acid-modified HA methacrylate (HAMA-PBA) and boronate ester bond between catechin and phenylboronic acid (the formed HAMA-PBA/catechin hydrogel bio-adhesive was named as HMPC) (Figure 3A) [57]. As a natural polyphenol, catechin has significant antioxidant activity, which effectively scavenges ROS and relieves oxidative stress. Under UV light, HMPC hydrogel was formed at irregular wound sites, and the cleavage of the boronate ester bond led to the release of catechins under high blood glucose conditions. In vitro and in vivo experimental results confirmed that the biocompatible HMPC hydrogel bio-adhesive could reduce the inflammatory response, promote neovascularization, and accelerate collagen deposition. 

In addition, HA derivatives have been used to construct hydrogel bio-adhesives to control inflammation and modulate the immune cell function of chronic and highly inflamed wounds. Carboxyl groups and hydroxyl groups on the HA molecular chains are able to be chemically modified to introduce other functional groups. For example, Franz and colleagues designed a high-sulfated HA (sHA)/collagen hydrogel bio-adhesive to modulate inflammatory macrophage activity (Figure 3B) [94]. Moreover, the binding of sHA and the protein network formed by collagen could control the differentiation of monocytes into macrophages during the inflammatory stage and activate the function of regulatory macrophages. In a skin wound model of diabetic mice, the hydrogel bio-adhesive not only down-regulated inflammation but also promoted neovascularization and pro-regenerative macrophage activation.

Oxidized HA (OHA), which inherits the fascinating physical and chemical properties of HA, has also been recruited to design hydrogel bio-adhesives [125]. In a study by Han et al., OHA was cross-linked with CMCS via Schiff base reaction to form a biocompatible and biodegradable hydrogel bio-adhesive (CMCS-OHA) for hemostasis [92]. The aldehyde groups of OHA could undergo Schiff base reaction with the amino groups of tissue and CMCS to achieve adhesion and cohesion simultaneously. This hydrogel bio-adhesive is demonstrated to have a good hemostatic property and wound healing effect. In another study, Chen et al. developed a series of injectable hydrogel bio-adhesives based on N-succinyl CS (NSC) and OHA, in which Ca^2+^ and four-arm amine-terminated poly-(ethylene glycol) (4-arm-PEG-NH_2_) were introduced to improve the bioactivity and mechanical property, respectively [93]. Similarly, the hydrogel bio-adhesives were formed by Schiff bases.

### 3.3. Alginate

**Figure 4:**
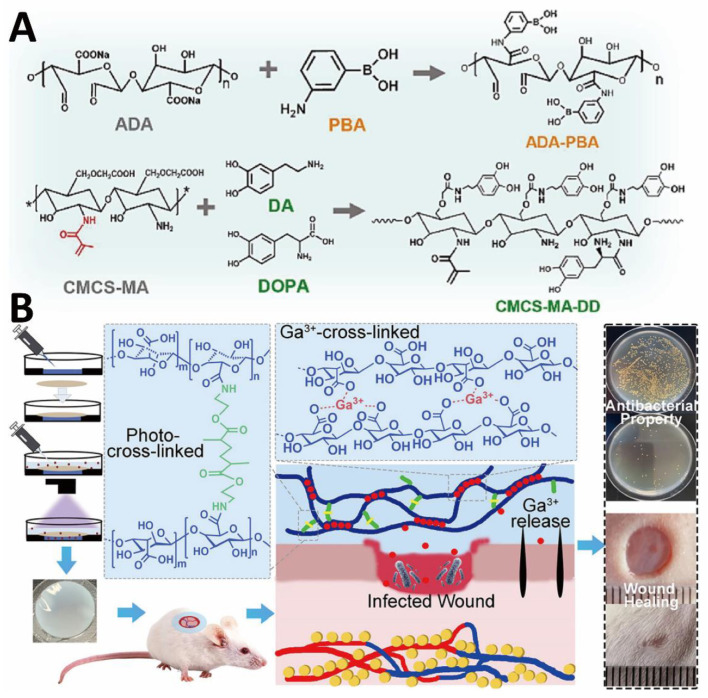
Alginate-based hydrogel bio-adhesives. (**A**) Preparation process of major components in H(P+T). Reprinted with permission from ref [96]. Copyright 2022 Elsevier. (**B**) Formation mechanism of double cross-linked alginate-based hydrogel bio-adhesive. It also demonstrated that the hydrogel bio-adhesive could effectively promote the healing of infected wounds. Reprinted with permission from ref [97]. Copyright 2022 American Chemical Society.

Alginate is a negatively charged natural polysaccharide that derives from brown algae cell walls and some bacterial strains [126]. Its linear sugar chain is composed of (1–4) β-D-mannuronic acid (M) and (1–4) α-L-guluronic acid (G) repeating units [127,128]. The good biocompatibility, hydrophilicity, moisturizing abilities, abundant sources, and controlled gelation properties make alginate a good candidate for wound healing hydrogel bio-adhesives. The alginate-based hydrogel bio-adhesives are able to absorb wound exudate and increase the concentration of blood cells and coagulation factors, which effectively accelerate hemostasis [129]. The carboxyl groups of alginates are able to chelate with Ca^2+^ to rapidly form calcium alginate hydrogel. In this hydrogel, the negative charge and Ca^2+^ were able to promote platelet aggregation, activate and accelerate the coagulation cascade, and further improve hemostatic efficiency [43]. DA is commonly used to modify alginate to confer superior adhesion and antioxidant activity. Furthermore, oxidized alginates and double-bond-modified alginates have also received increasing attention recently [130]. For example, Han et al. prepared a DA-grafted oxidized sodium alginate (OSA) hydrogel bio-adhesive (OSA-DA) via Schiff base reaction and oxidation of sodium periodate [95]. Compared with pristine alginate, OSA has good biodegradability and optimized adhesion property. Furthermore, such a biocompatible OSA-DA could effectively promote the migration of human umbilical vein endothelial cells (HUVECs). In a full-thickness excision chronic diabetic wound model, OSA-DA reduced inflammation, promoted neovascularization, and improved collagen deposition. In another study, Xie et al. developed a biocompatible multifunctional hydrogel bio-adhesive (H(P+T)) composed of CMCS, OSA, and tannic acid (TA). H(P+T) contained dynamic Schiff base bonds, dynamic boronate ester bonds, hydrogen bonds, and light-triggered covalent bonds [96]. To realize multiple cross-linking, catechol groups and methacrylates were modified on CMCS, and boron phenyl groups were modified on OSA (Figure 4A). As a common cross-linking agent, TA was added to the resulting derivatives and introduced hydrogen bonds. Compared with commercially available gels, H(P+T) showed sufficient mechanical strength, enhanced bio-adhesion, self-healing property, antioxidant, antibacterial, and hemostatic activities.

As reported, the chelation between alginate and divalent or polyvalent metal cations will form hydrogels with “eggshell” structures [131]. However, the mechanical properties and stabilities of such hydrogels are insufficient due to the rapid and uneven gelation. Therefore, additional cross-linking strategies were carried out to improve their mechanical strengths. For instance, Gu et al. designed a gallium ion (Ga^3+^) and light double-crosslinked alginate-based hydrogel bio-adhesive for the treatment of infected wounds and chronic inflammation (Figure 4B). Ga^3+^ was chelated with alginates to form the hydrogel bio-adhesives and effectively kill bacteria by disrupting bacterial iron metabolism through the “Trojan Horse” trick [97]. In another study, acrylate groups were modified into the alginate molecular chains to introduce photo-crosslinking and improve the mechanical properties of the hydrogel bio-adhesives. During wound healing in severe trauma, the formation of keloids and hypertrophic scars has adverse physiological and psychological effects on patients [132]. To address this issue, Lang and colleagues designed a bilayer thiolated alginate (SA-SH)/PEG diacrylate (PEG-DA) hydrogel bio-adhesive loaded with small extracellular vesicles (sEVs) [98]. By adjusting the ratios of SA-SH and PEG-DA, the degradation rates of the upper and lower layers of the hydrogel bio-adhesives could be altered to control the rational release of sEVs. During the inflammatory and proliferative phases of wound healing, sEVs released from the lower layers could promote the migration and proliferation of endothelial cells (ECs) and fibroblasts, neovascularization, and collagen deposition. In addition, during the later stage of the proliferation and remodeling, sEVs released from the upper layers were able to suppress excessive neovascularization and collagen deposition, thereby reducing the scar formation. 

### 3.4. Cellulose

**Figure 5:**
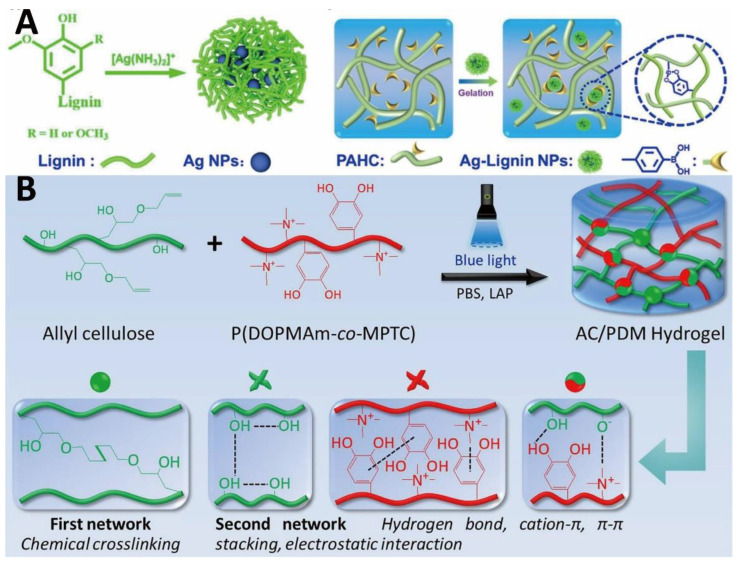
Cellulose-based hydrogel bio-adhesives: (**A**) formation illustration of Ag-Lignin NPs and HPC-based hydrogel bio-adhesive. Reprinted with permission from ref [99]. Copyright 2021 American Chemical Society. (**B**) Schematic of hydrogel formation under blue light and illustration of the double-network structure. Reprinted with permission from ref [100]. Copyright 2021 Elsevier.

D-glucose repeating units are linked by β-1,4 glycosidic bonds to form cellulose [133]. Cellulose has excellent biocompatibility and sufficient mechanical strength. However, cellulose is insoluble and undegradable in water and general organic solvents [133]. To date, various chemical strategies such as etherification and esterification have been used to improve the solubility of cellulose [134,135]. Carboxymethyl cellulose (CMC), which is able to coordinate with Fe^3+^, activate coagulation factors, and accelerate platelet aggregation, is the most common derivative of cellulose [136,137,138]. Moreover, oxidized cellulose with improved biodegradability is able to absorb wound exudate, aggregate blood cells, concentrate clotting factors, and promote fibrin clot formation to accelerate hemostasis [139]. Owing to its high biocompatibility, cellulose-based hydrogel bio-adhesives are widely used to load and release various substances to treat wounds. For example, a cellulose-chalcone hydrogel bio-adhesive containing multi-walled carbon nanotubes (MWCNTs) was developed, which could effectively and sustainably release bioactive compounds to fight against bacteria and promote wound healing [140]. In another research, Chen et al. prepared a multifunctional hydrogel bio-adhesive based on hydroxypropyl cellulose (HPC) and lignin. Phenylboronic acid-modified HPC was formed from HPC and 4-carboxyphenylboronic acid by esterification reaction [99]. Due to the existence of phenolic hydroxyl groups and methoxyl groups in the lignin molecular chains, the hydrogel bio-adhesive showed good adhesion and antioxidant properties. Ag^+^ was reduced into Ag nanoparticles (Ag NPs) by reductive lignin to confer the hydrogel bio-adhesive with strong antibacterial properties and electrical conductivity (Figure 5A). In addition, the catechol groups of lignin could form reversible coordination bonds with Ag NPs, and dynamic boronic ester bonds with phenylboronic acids to achieve self-healing and injectability of the hydrogel bio-adhesive (Figure 5A). Further study demonstrated that this hydrogel bio-adhesive could arrest bleeding, inhibit bacteria, reduce inflammatory responses, promote M2 macrophage polarization, neovascularization, collagen deposition, and re-epithelialization. Inspired by mussel adhesion, Wu and colleagues developed a dual-network biocompatible hydrogel bio-adhesive composed of cellulose and 3,4-dihydroxyphenylalanine (DOPA)-cationic copolymer for hemostasis and wound repair in incompressible and irregularly shaped wounds [100]. The unsaturated bonds were introduced into the cellulose ether molecular chains, and the rapid gelation was induced by a blue light (405 nm), which has better safety and tissue penetration than UV light (Figure 5B). A rapid and robust bio-adhesion was produced from the π-π stacking, cation-π interactions, metal-ligand coordination, and numerous hydrogen bonds between DOPA and biological tissues. Furthermore, DOPA-cationic polymers were able to impart enhanced adhesion, and excellent antibacterial and hemostatic activities to the hydrogel bio-adhesive. In a full-thickness skin defect model, the cellulose-based hydrogel bio-adhesive promoted neovascularization, collagen deposition, and granulation tissue formation.

### 3.5. Dextran

**Figure 6:**
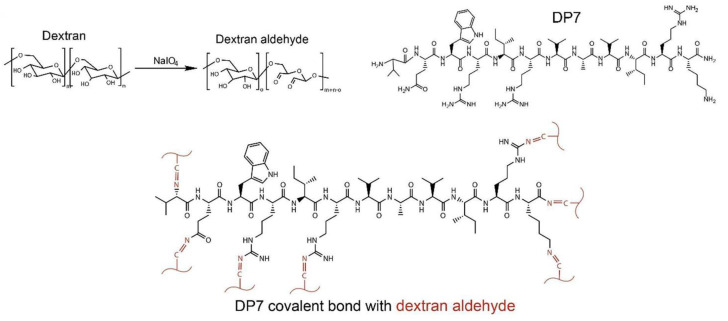
Dextran-based hydrogel bio-adhesive: chemical reaction mechanisms in hydrogel bio-adhesive. Reprinted with permission from ref [101]. Copyright 2022 Elsevier.

Dextran is derived from microorganisms and is an electrically neutral polysaccharide. Dextran is composed of α-1,6-linked glucose monomers and α-1,3 branched chains and has excellent biocompatibility and biodegradability. The adjacent hydroxyl groups on dextran are able to partially oxidize into aldehyde groups. Through the Schiff base reaction, the oxidized dextran (ODEX) not only cross-linked with polymers containing amino groups but also reacted with amino groups of tissues to afford the adhesion [141,142]. In a recent study, Yang et al. developed a pH-sensitive ceftazidime-loaded hydrogel bio-adhesive using ODEX and antimicrobial peptide DP7 (VQWRIRVAVIRK) to inhibit multidrug-resistant bacteria [101]. ODEX formed hydrogel bio-adhesive with DP7 that possessed amino groups via Schiff bases (Figure 6). The low pH of infected wounds could disrupt the Schiff base bonds and allow the local controlled release of ceftazidime. Due to the broad-spectrum antibacterial and pro-healing activities of DP7, as well as the bio-adhesion of ODEX, the hydrogel bio-adhesive could effectively promote the healing of infected wounds. It was worth mentioning that the hydrogel bio-adhesive is able to promote scar-free wound repair, and hydrogel bio-adhesive-treated wounds had no prominent scar tissues after healing.

### 3.6. Konjac Glucomannan (KGM)

**Figure 7:**
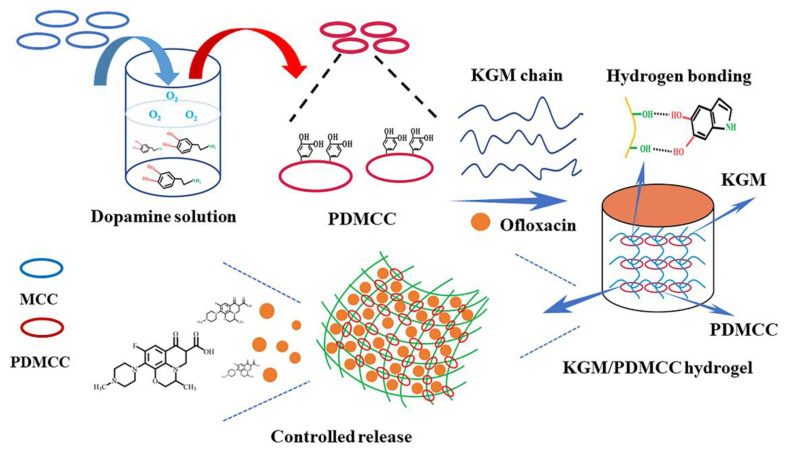
KGM-based hydrogel bio-adhesive: preparation diagram of KGM/PDMCC hydrogel bio-adhesive. Reprinted with permission from ref [144]. Copyright 2018 Elsevier.

KGM, composed of D-mannose and D-glucose linked by β-1,4 glycosidic bonds in a molar ratio of 1.4–1.6:1, is a natural electrically neutral polysaccharide derived from the tuber of konjac. KGM is biocompatible, biodegradable, water-soluble, and has excellent gel properties. In addition, it is capable of exerting immunomodulatory effects to promote wound healing. For example, KGM is able to promote the secretion of anti-inflammatory factors such as Interleukin-10 (IL-10) from macrophages and accelerate the transformation of macrophages from pro-inflammatory M1 type to anti-inflammatory M2 type [143]. KGM-based hydrogel bio-adhesives have good potential for controlled drug release. Wu and colleagues designed an ofloxacin-loaded hydrogel bio-adhesive based on KGM and microcrystalline cellulose (MCC) that exhibited pH-sensitive release and low initial burst release (Figure 7) [144]. Due to the high crystallinity and cellulose content of MCC, it had been used as a reinforcing component for hydrogel bio-adhesive. Through the oxidative polymerization of DA, MCC was functionalized to form PDMCC. Strong intermolecular hydrogen bond interaction existed between KGM and PDMCC. As a consequence, the introduction of PDMCC increased the cross-linking density and improved the drug loading efficiency, self-healing, and mechanical properties of the hydrogel bio-adhesive. Moreover, KGM is able to be chemically modified to meet different needs. A CMCS/collagen peptide (COP)/oxidized KGM (OKGM) hydrogel bio-adhesive was prepared by Fan et al. [102]. In this study, KGM was oxidized by sodium periodate to form OKGM, which was then cross-linked with CMCS/COP via the Schiff base reactions. The final composite hydrogel bio-adhesive was demonstrated to have good biocompatibility, water retention capacity, and excellent mechanical property, which could effectively promote wound healing.

### 3.7. Chondroitin Sulfate

**Figure 8:**
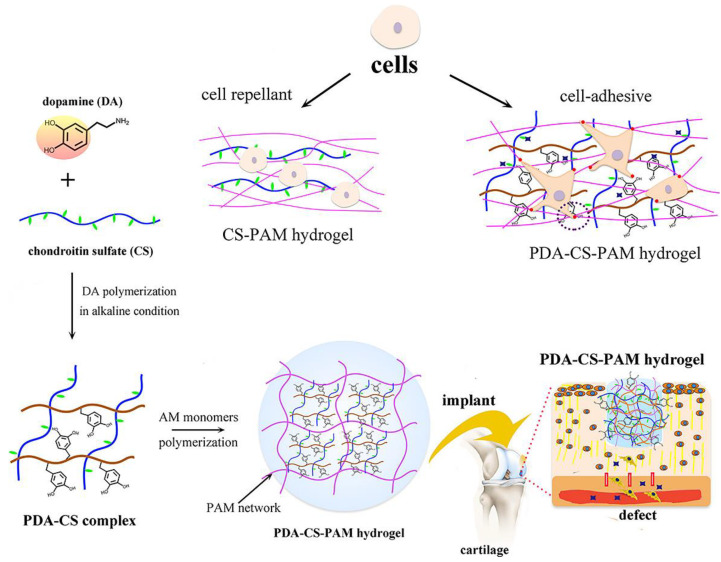
Chondroitin sulfate-based hydrogel bio-adhesive: preparation process and application of mussel-inspired chondroitin sulfate-based hydrogel bio-adhesives. Reprinted with permission from ref [148]. Copyright 2022 American Chemical Society.

Chondroitin sulfate belongs to the sulfated glycosaminoglycans, mainly composed of β-1,3-linked-N-acetyl-galactosamine and β-1,4-linked-glucuronic acid sugar residues [145]. Chondroitin sulfate has inherent biodegradability, hydrophilicity, mucoadhesive property, immunomodulatory, antioxidant, and anti-inflammatory activities [146]. Moreover, it has been found that chondroitin sulfate is able to reduce the levels of nuclear factor-κβ (NF-κβ), matrix-degrading enzymes, and interleukin-1β (IL-1β) and downregulate the inflammatory response [146]. Chondroitin sulfate is able to be oxidized by sodium periodate to generate active -CHO in the D-glucuronic acid repeating units. With the above features, chondroitin sulfate-based hydrogel bio-adhesives are now widely used to treat tissue damage. For example, an in situ injectable hydrogel bio-adhesive based on N, O-CMCS, and oxidized chondroitin sulfate was developed by He et al. for efficient wound healing [103]. N, O-CMCS, and oxidized chondroitin sulfate gelled via Schiff bases. The formed hydrogel bio-adhesive was biocompatible and biodegradable, which had strong adhesion, self-healing, antibacterial, and hemostatic activities. Furthermore, chondroitin sulfate has the therapeutic potential to promote cartilage regeneration [147]. Inspired by mussels, Lu et al. developed a PDA-chondroitin sulfate-PAM hydrogel bio-adhesive for cartilage repair (Figure 8) [148]. PDA with abundant catechol groups self-assembled with chondroitin sulfate to form a PDA-chondroitin sulfate complex with good cellular affinity and strong adhesion. The final PDA-chondroitin sulfate-PAM hydrogel bio-adhesive exhibited suitable mechanical and adhesive strength and could support the cartilage regeneration.

### 3.8. Other Polysaccharides

Other polysaccharides such as pullulan and curdlan-based hydrogel bio-adhesive have also been studied in wound healing. As a microbial exopolysaccharide, pullulan is produced by *Aureobasidium pullulans* in submerged fermentation [149]. Sohail and co-workers reported a HA and Pullulan-based injectable hydrogel. After being loaded with curcumin, the hydrogel could increase angiogenesis, potentiate reepithelization, and collagen deposition at a wound microenvironment to endorse a healing cascade. Such a biocompatible and curcumin-laden hydrogel was proved to potentiate wound healing in a streptozotocin-induced diabetic rat model by promoting 93% of wound closure [150]. In another study, the mechanically robust, self-healing, injectable, and biodegradable pullulan-PEG hydrogel was designed by covalent crosslinking of 8-arm PEG hydrazine and oxidized pullulan using dynamic and pH-sensitive hydrazone linkages. After being loaded with dexamethasone, the hydrogel exhibited promising antioxidant and anti-inflammatory properties and promoted the proliferation and 3D encapsulation of murine osteoblast precursor cells [151]. Curdlan is an unbranched, bacterial β-1,3-glucan, which possesses beneficial rheological, chemical, and biological properties [152]. Recently, toxicity-free curdlan/PDA composite hydrogels were designed and used for periodontal antibacterial treatment by combining antimicrobial and photothermal effects simultaneously [153].

## 4. Conclusions and Future Outlook

Wound healing is a dynamic and complex process involving multiple cells, cytokines, signaling pathways, and related factors [154]. Hydrogel bio-adhesives have shown great potential in the healing of various types of wounds. Adhesion and cohesion mechanisms of hydrogel bio-adhesives are crucial for adhesive strength, and rational design strategies that may achieve a balance between adhesion and cohesion are necessary. With the deepening of research and the expansion of clinical needs, the functionalities and therapeutic effects of hydrogel bio-adhesives are further enhanced. This review summarizes the attractive properties of various hydrogel bio-adhesives that have been developed, such as adhesion, self-healing, stimuli-responsive property, electrical conductivity, hemostatic, antibacterial, antioxidant, and anti-inflammatory activities, etc. In addition, as delivery systems, hydrogel bio-adhesives should be able to load and slowly release therapeutic drugs to the wound sites [155]. Furthermore, in the design of hydrogel bio-adhesives, biocompatibility, and biodegradability should be considered. Most polysaccharides have inherent biocompatibility and biodegradability, unique bioactivity, great healing-promoting activity, etc., and have been widely used in the manufacture of hydrogel bio-adhesives. The control of the biodegradability of hydrogel bio-adhesives is essential since their degradation ratios should be kinetically matched with the wound-healing process. The selection, chemical modification, and cross-linking of polysaccharides determine the functionality and properties of the final hydrogel bio-adhesives, thereby driving their utility and expanding their range of applications.

Although polysaccharide-based hydrogel bio-adhesives have been developed with various functions, they are challenging to satisfy the entire repair process in dynamically changing wounds. For instance, therapeutic drugs and cytokines are only required at specific stages; yet, for some stages, they may even hinder the healing process [33]. Given the diversity of wounds, hydrogel bio-adhesives need to meet the unique requirements of various wounds, and their personalized customization is one of the future research directions. For example, the pH of normal wounds is acidic, while the pH of chronic wounds is alkaline [156]. Moreover, how to achieve the on-demand removal property of adhesive hydrogel bio-adhesives remains a major problem. Preclinical animal studies are critical, and mice and rats are chosen as routine model organisms. Nevertheless, their wound-healing processes differ from humans, leading to biased conclusions. Therefore, additional suitable standardized animal models, for example, large laboratory animal models, are necessary to determine the efficacy and safety of the final hydrogel bio-adhesives [157]. Furthermore, the mechanical strength, wet adhesion strength, and gel time of the hydrogel bio-adhesives for hemostasis need to be considered [158]. Recently, hydrogel bio-adhesives capable of monitoring and managing wounds have appeared and showed a trend of intelligence [159]. The combination of wound monitoring and telemedicine, which enables remote-controlled treatment, is a promising research area in the future. Although the diversified modification, component selection, and ratio adjustment of polysaccharides may improve various functions of hydrogel bio-adhesives to a certain extent, they are still far from clinical applications. In addition, their practicality, ease of design and fabrication, and long-term storage also need to be considered. Furthermore, a bright future can be foreseen for the effective treatment of wounds via multidisciplinary strategies such as the integration of nanoparticles with hydrogel bio-adhesives [160]. Ultimately, there is a long way to go before the development and commercialization of polysaccharide-based hydrogel bio-adhesives for perfect wound healing.

## Figures and Tables

**Table 1:** Role in wound healing and derivatives of various polysaccharides.

Polysaccharides	Role in Wound Healing	Derivatives
CS	Enhances platelet function; and promotes the adhesion and aggregation of platelets, red blood cells, and proteins. Mucoadhesive, antibacterial, regulates inflammatory mediator secretion, enhances the function of inflammatory cells; and promotes fibroblast proliferation, neovascularization, collagen deposition, and granulation tissue formation.	Carboxymethyl CS [86], quaternized CS [87], thiolated CS [88], methacrylated CS [89], catechol-modified CS [89,90], hydrophobically modified CS [91]
HA	Tissue adhesion, blood concentration, keeps the wound moist, promotes cell migration and proliferation, reduces the infiltration of inflammatory cells, regulates growth factor activity, stimulates neovascularization, controls collagen deposition, and improves granulation tissue formation and re-epithelialization.	Oxidized HA [92,93], methacrylated HA [57], sulfated HA [94]
Alginate	Tissue adhesion, blood concentration, keeps the wound moist, and promotes fibroblast proliferation and granulation tissue formation.	Oxidized alginate [95,96], methacrylated alginate [97], thiolated alginate [98]
Cellulose	Blood concentration, activates coagulation factors, and promotes platelet aggregation.	Hydroxypropyl cellulose [99], methacrylated cellulose [100]
Dextran	Keeps the wound moist, accelerates platelet adhesion, and promotes cell migration and proliferation.	Oxidized dextran [101]
KGM	Regulates immunity, promotes anti-inflammatory factors secretion, and macrophage M2-type polarization.	Oxidized KGM [102]
Chondroitin sulfate	Mucosa adhesion, regulates immunity, antioxidant, reduces inflammation, and promotes cartilage regeneration.	Oxidized chondroitin sulfate [103]

## Data Availability

Not applicable.

## Abbreviations

CS	Chitosan
HA	Hyaluronic acid
KGM	Konjac glucomannan
ECM	Extracellular matrix
ROS	Reactive oxygen species
DNA	Deoxyribonucleic acid
DA	Dopamine
DPPH	α, α-Diphenyl-β-picrylhydrazine
ABTS	2,2-Diazo-bis (3-ethyl-benzothiazole-6-sulfonic acid) diammonium salt
GFs	Growth factors
ADSCs	Adipose-derived stem cells
BMSCs	Bone marrow mesenchymal stem cells
HMECs	Human microvascular endothelial cells
EGF	Epidermal growth factor
FGF	Fibroblast growth Factor
VEGF	Vascular endothelial growth factor
KGF	Keratinocyte growth Factor
PEG	Polyethylene glycol
PI3 kinase/Pten	Phosphoinositide 3-kinases/phosphatase and tensin homologue
CMCS	Carboxymethyl chitosan
QCS	Quaternized chitosan
PAM	Polyacrylamide
CQDs	Carbon quantum dots
CS-TBA	Chitosan-4-thiobutylamidine
β-GP	β-Glycerophosphate disodium
G’	Storage modulus
G”	Loss modulus
rGO@PDA	Polydopamine-coated reduced graphene oxide
HAMA-PBA	Phenylboronic acid-modified hyaluronic acid methacrylate
sHA	Sulfated hyaluronic acid
OHA	Oxidized hyaluronic acid
NSC	N-succinyl chitosan
4-arm-PEG-NH2	Four-arm amine-terminated poly-(ethylene glycol)
OSA	Oxidized sodium alginate
HUVECs	Human umbilical vein endothelial cells
TA	Tannic acid
SA-SH	Thiolated alginate
PEG-DA	Polyethylene glycol diacrylate
sEVs	Small extracellular vesicles
ECs	Endothelial cells
CMC	Carboxymethyl cellulose
MWCNTs	Multi-walled CNTs
HPC	Hydroxypropyl cellulose
DOPA	3,4-Dihydroxyphenylalanine
ODEX	Oxidized dextran
MCC	Microcrystalline cellulose
COP	Collagen peptide
OKGM	Oxidized konjac glucomannan
NF-κβ	Nuclear factor-κβ
IL-1β	Interleukin-1β
PDA	Polydopamine
IL-10	Interleukin-10
